# Supplementary material for: HIVprotI: an integrated web based platform for prediction and design of HIV proteins inhibitors
Source: J Cheminform. 2018 Mar 9;10:12. doi: 10.1186/s13321-018-0266-y (PMC5845081; doi:10.1186/s13321-018-0266-y)
Supplement: Supplementary file 2 — Additional file 2. Source code of HIVProtI web server. [file 13321_2018_266_MOESM2_ESM.zip › HIVprotI_Source-code/hivprotI/weka-3-6-6/doc/weka/gui/visualize/plugins/VisualizePlugin.html]

VisualizePlugin


---


|  |  |  |
| --- | --- | --- |
| **Overview** \| **Package** \| **Class** \| **Tree** \| **Deprecated** \| **Index** \| **Help** \| **Weka's home** | |  |
| PREV CLASS   NEXT CLASS | **FRAMES**    **NO FRAMES**     **All Classes** |  |
| SUMMARY: NESTED \| FIELD \| CONSTR \| METHOD | DETAIL: FIELD \| CONSTR \| METHOD |  |


---


## weka.gui.visualize.plugins Interface VisualizePlugin

---

``` public interface VisualizePlugin ```

Interface implemented by classes loaded dynamically to
visualize classifier results in the explorer.

**Version:**
:   $Revision: 1.1 $

**Author:**
:   Jeffery Grajkowski (grajkows@cs.ualberta.ca)

---

| **Method Summary** | |
| --- | --- |
| `java.lang.String` | `getDesignVersion()`             Get the specific version of Weka the class is designed for. |
| `java.lang.String` | `getMaxVersion()`             Get the maximum version of Weka, exclusive, the class is designed to work with. |
| `java.lang.String` | `getMinVersion()`             Get the minimum version of Weka, inclusive, the class is designed to work with. |
| `javax.swing.JMenuItem` | `getVisualizeMenuItem(FastVector preds, Attribute classAtt)`             Get a JMenu or JMenuItem which contain action listeners that perform the visualization, using some but not necessarily all of the data. |

**Method Detail**

### getVisualizeMenuItem

```
javax.swing.JMenuItem getVisualizeMenuItem(FastVector preds,
                                           Attribute classAtt)
```

:   Get a JMenu or JMenuItem which contain action listeners
    that perform the visualization, using some but not
    necessarily all of the data. Exceptions thrown because of
    changes in Weka since compilation need to be caught by
    the implementer.

    :   **Parameters:**: `preds` - predictions: `classAtt` - class attribute **Returns:**: menuitem for opening visualization(s), or null to indicate no visualization is applicable for the input **See Also:**: `NoClassDefFoundError`, `IncompatibleClassChangeError`

---


### getMinVersion

```
java.lang.String getMinVersion()
```

:   Get the minimum version of Weka, inclusive, the class
    is designed to work with. eg: `3.5.0`

---


### getMaxVersion

```
java.lang.String getMaxVersion()
```

:   Get the maximum version of Weka, exclusive, the class
    is designed to work with. eg: `3.6.0`

---


### getDesignVersion

```
java.lang.String getDesignVersion()
```

:   Get the specific version of Weka the class is designed for.
    eg: `3.5.1`


---


|  |  |  |
| --- | --- | --- |
| **Overview** \| **Package** \| **Class** \| **Tree** \| **Deprecated** \| **Index** \| **Help** \| **Weka's home** | |  |
| PREV CLASS   NEXT CLASS | **FRAMES**    **NO FRAMES**     **All Classes** |  |
| SUMMARY: NESTED \| FIELD \| CONSTR \| METHOD | DETAIL: FIELD \| CONSTR \| METHOD |  |


---
